# Efficiency of Cell Therapy to GC-Induced ONFH: BMSCs with Dkk-1 Interference Is Not Superior to Unmodified BMSCs

**DOI:** 10.1155/2018/1340252

**Published:** 2018-05-22

**Authors:** Wei Zhun, Li Donghai, Yang Zhouyuan, Zhao Haiyan, Kang Pengde

**Affiliations:** Department of Orthopaedics Surgery, West China Hospital, Sichuan University, No. 37 Wainan Guoxue Road, Chengdu, China

## Abstract

Glucocorticoid-induced osteonecrosis of the femoral head (ONFH) is a hip disorder, and it threatens patients who require megadose of steroid therapies. Nowadays, no valid therapies can reverse the development of GC-induced ONFH once it occurs. Stem cell therapy to GC-induced ONFH would be a promising choice. Although the pathogenesis of GC-induced ONFH is not yet fully clear, Dickkopf-1 (Dkk-1) upregulated by excessive GC use, which hinders the canonical Wnt pathway, could be an explanation. Thus, the aim of the present work lies in investigating the efficiency of the allograft bone marrow stem cells (BMSCs) with Dkk-1 interference in preventing the progression of the GC-induced ONFH. Lentivirus-meditated Dkk-1 RNAi was introduced into BMSCs which was exposed to dexamethasone (10^−6^ mol/L) in vitro. This interference blocked Dkk-1 overexpression by GC and afterwards prompted the transduction of Wnt/*β*-catenin in which the Runx2 and PPAR*γ* were upregulated and downregulated, respectively. Thus, the osteogenesis was promoted while adipogenesis was inhibited. In vivo, GC-induced ONFH rats were treated by allotransplantation of BMSCs with Dkk-1 interference, and the progression of the disease was prevented. However, the effects were not significantly superior to treatment with nongenetically modified or normal BMSCs.

## 1. Introduction

Excessive glucocorticoid (GC) use is one of the etiologies of osteonecrosis of the femoral head (ONFH). Although the pathophysiological mechanism of GC-induced ONFH remains unclear, reduced osteogenesis due to an aberrant decrease in the bone marrow mesenchymal stem cell (BMSC) pool may play a role [1, 2]. Thus, in addition to traditional procedures (e.g., core decompression, osteotomies, and bone grafting), BMSC implantation has been proposed as a potential therapy for GC-induced ONFH, especially in the early stages, with the aim of delaying or even obviating total hip arthroplasty (THA) [3–7].

BMSC implantation is often combined with core decompression. BMSC implantation involves the following steps [8, 9]: (1) harvesting of autologous or allogeneic BMSCs; (2) canonical core decompression; and (3) implantation of BMSCs in necrotic areas through the decompression tunnel. Although improved outcomes have been observed for BMSC treatment compared with canonical core decompression, concerns about the remaining osteogenic capacity or proliferation of BMSCs in the repair of GC-induced ONFH [10–12] have limited the widespread use of this treatment.

We and others [13–16] have shown that Dickkopf-1 (Dkk-1), which is upregulated by GC use, suppresses the transduction of the Wnt/*β*-catenin cascade in BMSCs and promotes their differentiation into adipocytes, implying a potential role of Dkk-1 in the pathogenesis of GC-induced ONFH. Wang and colleagues [17] demonstrated that Dkk-1 knockdown by an antisense oligonucleotide enhances the osteogenesis and proliferation of osteoblasts and attenuates adipocytic differentiation. Additionally, Tang et al. [18] and Hang et al. [19] enhanced the recovery of BMSCs in ONFH by introducing an exogenous BMP-2 or VEGF gene. These genes are responsible for osteoblastic lineage differentiation or angiogenesis, respectively. Based on these previous findings, we hypothesized that interference with the expression of Dkk-1 in BMSCs would arrest the progression of GC-induced ONFH by simultaneously suppressing the adipogenesis and enhancing the osteogenesis of BMSCs.

## 2. Methods

### 2.1. Isolation, Culture, and Identification of Primary BMSCs

One-week-old Sprague-Dawley rat pups were sacrificed sterilely, and both bilateral femurs and tibias were isolated cleanly. The isolated tissues were rinsed with sterile low-glucose Dulbecco's Modified Eagle Medium (DMEM), and the epiphyses and growth plates of the femurs and tibias were dissected on sterile dishes. The marrow cavities were flushed 3-4 times by inserting a needle attached to a 2 ml syringe containing low-glucose complete medium (low-glucose DMEM with 10% fetal bovine serum), and the resultant bone marrow suspensions were harvested into 15 ml tubes. After centrifugation at 1300 rpm for 5 minutes, the supernatants were removed, and the cells were suspended in complete medium (low glucose) in culture flasks and incubated in a humidified chamber at 37°C with 5% CO_2_. After 48 h of primary culture, nonadherent cells were removed by replacing the medium with fresh medium. The medium was replaced every 2 days thereafter. To verify the BMSCs, 3rd-generation cells were subjected to morphological observations, osteogenic and adipogenic induction, and phenotypic analyses.

### 2.2. Lentivirus-Meditated Dkk-1 RNAi Vector Construction and Analysis of Transfection Efficiency

The lentiviral vectors for Dkk-1 RNAi (sequence: GTACAAATCTGCCTGGCTT) and its negative control counterpart (sequence: TTCTCCGAACGTGTCACGT) were designed and constructed by GeneChem Inc. (see the flowchart in Figure 2 of the supplementary data). The transfection efficiency was optimal when viral dosage (defined by multiplicity of infection, MOI) reached to 25. (MOI = 25) (Figure 3, supplementary data).

### 2.3. In Vitro Lentivirus-Meditated Dkk-1 RNA Interference and Glucocorticoid Treatments

When their primary progenitors were passaged to the 3rd generation, the subcultured BMSCs were divided into 4 groups and seeded onto separate 6-well plates: the (1) transfection group (TG); (2) GC group (GG); (3) negative group (NG); and (4) blank group (BG). When the cells in each well of the 4 plates reached 30% confluence, TG and NG were transfected with the Dkk-1 RNAi vector or negative control vector, respectively. After 12 h and 48 h, the medium in each plate was renewed with low-glucose complete medium. At 96 h after transfection, the medium in TG, GG, and NG was replaced with fresh medium containing 10 *μ*M dexamethasone (Dex), whereas in BG, the medium was renewed as before. All groups were consecutively cultured for 14 days before subsequent analysis.

### 2.4. Real-Time qPCR Analysis

A two-step PCR method was applied. Briefly, total RNA was isolated from the BMSCs using a Bioteke kit (Bioteke Corporation), followed by cDNA synthesis using an iScript™ cDNA synthesis kit (Bio-Rad). The primers for *Ctnnb*1, *Runx*2, *Dkk-*1, *Gsk*3*b*, *Pparg*, and *Gapdh* (primer sequences are listed in Table 1 of the supplementary data) were synthesized by Beijing Tsingke Biological Technology. The qPCR followed the protocol for the SsoFast EvaGreen supermix kit (Bio-Rad) with the following cycling parameters in the LightCycler® 96 System: predenaturation at 95°C for 30 s; 40 cycles of denaturation at 95°C for 5 s and annealing/extension at 50–58°C for 10 s (40 cycles); and melting curve analysis at 65–95°C, 10 sec/step. The fold change in relative mRNA expression was calculated by the 2^−ΔΔCt^ method.

### 2.5. Western Blotting Analysis

Cells were lysed in RIPA buffer with protease inhibitor cocktail on ice for 30 min, followed by centrifugation at 4°C and 1200 rpm for 30 min. The supernatant was stored at −80°C. The protein concentration was determined by the BCA method, and equal amounts of protein were separated by SDS-PAGE. For Western blotting, the samples on the gel were transferred to PVDF membranes, and the membranes were washed with TBST for 5 min, followed by blocking in 5% BSA in TBST for 1 h and washing three times with TBST for 5 min each. The membranes were then incubated with primary antibodies (Runx2, PPAR*γ*-2, GSK-3*β*, *β*-catenin, Dkk-1, and GAPDH, all 1 : 1000 dilution) overnight at 4°C and washed 3 times with TBST for 5 min each before incubation with the secondary antibody at room temperature for 1 h. After washing the membranes with TBST three times for 5 min each, the samples on the membranes were visualized by ECL and scanned by Quantity One software (Bio-Rad).

### 2.6. Establishment of the GC-Induced ONFH Rat Model

A total of 85 adult SPF Sprague-Dawley rats (purchased from Chengdu Dossy Biological Technology Co. Ltd, weighting between 250 to 350 g) were intramuscularly (i.m.) injected with lipopolysaccharide (LPS) once at a dose of 10 *μ*g/kg. Methylprednisolone (MPS) was then i.m. injected at a dose of 20 mg/kg once daily for the next three days. To ensure survival, all rats received gentamicin solution (8 × 10^3^ U/d) by gavage for the next seven days, combined with i.m. injection of lansoprazole (1 mg/d). Thereafter, all rats were carefully housed and maintained for the next 6 weeks (protocol and results in Figures 4 and 5, supplementary data).

### 2.7. Cell Implantation into Femoral Heads

After 6 weeks, the remaining 80 rats (4 rats died during the period of establishing GC-induced ONFH model, and one was excluded to ensure equal group sizes) were randomly allocated to 4 groups: the control group, which was treated with normal saline (control); the therapeutic group, which was treated with Dkk-1 RNAi-modified BMSCs (LV-Dkk-1-RNAi-BMSCs, LDRM); the negative control group, which was treated with empty sequence-modified BMSCs (LV-ES-BMSCs, LEM); and the normal therapeutic group, which was treated with normal, unmodified BMSCs. Due to the small size of the rat femoral head, intrabone marrow injection was employed [20]. To perform the procedure, the animal was anesthetized using chloral hydrate, and the lower limb was disinfected. An incision was made in the center of the knee, and the skin was dissected bluntly layer-by-layer to open the knee cavity. A 2 ml needle was inserted into the intercondylar, penetrating the marrow cavity of the femur and approaching beneath the trochanter major. Then, 0.1 ml of BMSCs (10^7^ cells/ml) or normal saline mixed with heparin was injected; the marrow cavity was sealed with sterilized bone wax, and the skin was sutured. Gentamycin was administered in-feed for the next week to prevent further infection, and the rats were observed for another 7 weeks. After sacrifice, the bilateral femoral heads of all rats (except those used for frozen sections) were dissected, fixed in 4% paraformaldehyde, decalcified in EDTA solution for 3 weeks, washed with deionized water for 10 min, dehydrated in a gradient of ethyl alcohol, waxed, embedded, and sliced into 3 *μ*m thick sections. The protocol is illustrated in brief in Figure 4 in the supplementary data.

### 2.8. In Vitro and In Vivo Histological Analyses

Cell samples were stained with Oil Red O or alizarin red in vitro. The culture medium was aspirated completely from BMSCs incubated in 6-well plates, and the cells were rinsed 3 times with PBS and fixed in 4% paraformaldehyde for 10 min. After rinsing 3 times with PBS, the cells were incubated with Oil Red O solution (Sigma, USA) or alizarin red (Sigma, USA) at 37°C for 20 min. Excess solution was then removed by rinsing with distilled water, followed by staining with hematoxylin for 2 min. After rinsing again with distilled water, the cells were mounted in aqueous mounting medium and observed by phase contrast microscopy.

Frozen sections of the femoral heads of rats were also subjected to Oil Red O staining. The frozen slices were prepared and placed in absolute propylene glycol for 5 min. The slices were then stained with Oil Red O solution (Sigma, USA) for 20 min, followed by differentiation in 85% propylene glycol for 2 min. After rinsing 3 times with distilled water, the slices were stained with hematoxylin for 1 min, rinsed with distilled water, and mounted on a coverslip in aqueous mounting medium. All slices were observed by microscopy.

Hematoxylin and eosin (H&E) staining was performed on paraffin slices of the femoral heads of rats as follows: the slices were dewaxed in xylene, dehydrated in a gradient of ethyl alcohol, rinsed 3 times with distilled water, stained with hematoxylin, differentiated in 1% HCl ethanol for 30 s, washed with running tap water for 15 min, stained with eosin, washed with running tap water for 3 min, dehydrated again in a gradient of ethyl alcohol, vitrified in xylene, and finally mounted in resinene.

### 2.9. Immunohistochemical Analysis

Femoral head slices were dewaxed and hydrated, followed by antigen retrieval in sodium citrate (pH 6.0) at 95°C for 40 min. After cooling to room temperature, peroxidases were inactivated by incubating the slices in 3% H_2_O_2_ for 15 min. The slices were incubated in a humid atmosphere with diluted primary antibodies (Dkk-1, GSK-3*β*, *β*-catenin, PPAR*γ*2, and Runx2) at 37°C for 45 min, washed 3 times with PBS, and then incubated with biotin-conjugated secondary antibody at 37°C for 45 min. HRP and 100 *μ*l of DAB was then added. Staining was monitored under a microscope, and the reactions were terminated by the addition of distilled water. The slices were then stained with hematoxylin, dehydrated in a gradient of ethyl alcohol, vitrified in xylene, mounted in neutral resin, and evaluated by light microscopy in a 400-fold magnified field. Five fields were randomly selected and captured by Image-Pro Plus 6.0 to calculate the percentage of areas of positive expression.

### 2.10. Statistical Analyses

All statistical analyses were performed using SPSS 17.0 software, and differences were considered significant at *P* < 0.05. ANOVA with multiple comparisons was used to analyze all parametric data. The incidence of GC-induced ONFH in rats was assessed by *χ* ^2^ tests with multiple comparisons. All bar plots were prepared in ggplot2 (version 2.21).

## 3. Results

### 3.1. The Effects of GC Treatment and Lentivirus-Meditated Dkk-1 RNAi on the Osteogenesis and Adipogenesis of BMSCs In Vitro

**Figure 1:**
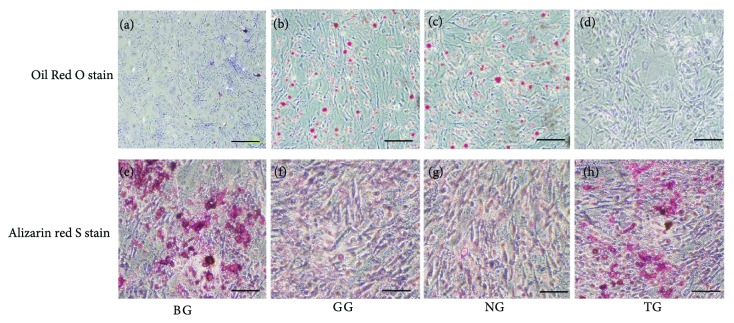
Oil Red O staining indicating the adipogenesis (red droplets for lipid, (a–d)) and alizarin red staining indicating osteogenesis (red nodules for mineralized calcium, (e–h)). No sign of red droplets in the blank group (BG, (a)) or the transfection group (TG, (d)) meant no lipid formation in the two groups. While in GC group (GG, (b)) and negative group (NG, (c)), significant red droplets suggested more lipid formation. On the other hand, BG and TG formed significantly more mineralized calcium than the GC and NG.

After consecutive 14-day cultivation of all BMSC groups, in vitro adipogenesis or osteogenesis was visualized by Oil Red O or alizarin red S staining, respectively. As shown in Figures 1(a)–1(d), larger areas of red staining indicated greater lipid accumulation and adipogenesis. Adipogenic differentiation was much lower in BG and TG than in GG and NG. Consistent with these results, more calcium formation was observed in BG and TG than in GG and NG, as demonstrated by the area of red staining (Figures 1(e)–1(h)), indicating greater osteogenesis in both BG and TG than in GG and NG.

### 3.2. In Vitro RT-PCR and Western Blotting Analyses of Dkk-1, GSK-3*β*, *β*-Catenin, Runx2, and PPAR*γ*

**Figure 2:**
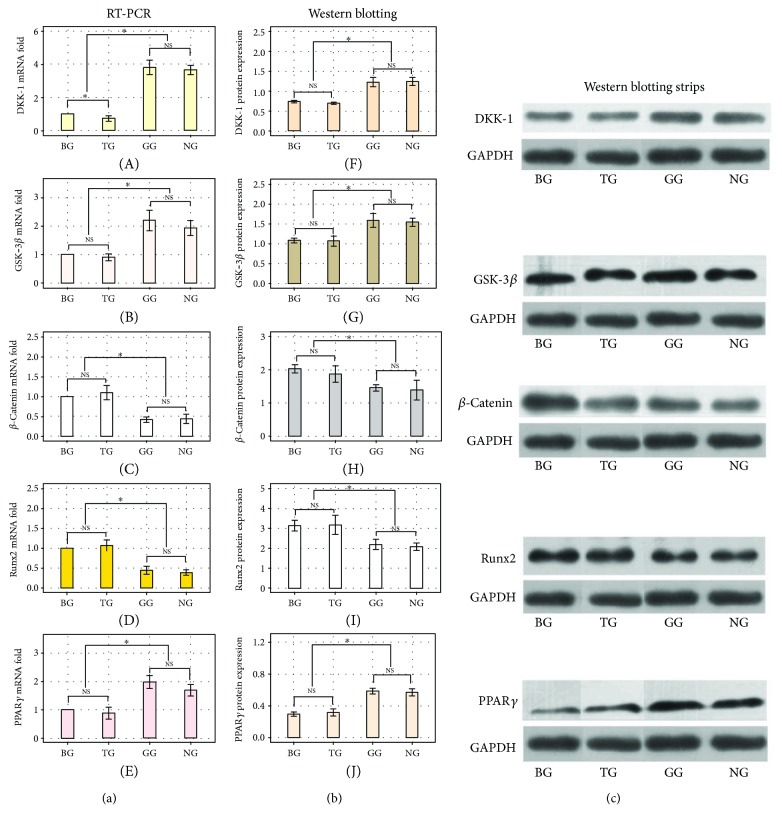
RT-PCR analyses (a) for mRNA levels of Dkk-1, GSK3*β*, *β*-catenin, Runx2, and PPAR*γ*. Dkk-1, the antagnosist of Wnt/*β*-catenin pathway, its mRNA level in TG were significantly lower than BG, GG or NG (*P* < 0.05) but not between GG and NG (*P* > 0.05). GSK3*β* mRNA in BG or TG was significantly lower (*P* < 0.05) than that of GG or NG, and mRNA of *β*-catenin in TG or BG was higher (*P* < 0.05) than that in GG or NG. Runx2 was higher (*P* < 0.05) in BG or TG than that in GG or NG while PPAR*γ* was lower (*P* < 0.05) in BG or TG than that in GG or NG. The mRNA level of GSK3*β*, *β*-catenin, Runx2, or PPAR*γ* was not significantly different between BG and TG (*P* > 0.05) or between GG and NG (*P* > 0.05). Western blotting analyses (b and c) of the expressions of Dkk-1, GSK3, *β*-catenin, Runx2, and PPAR*γ*. The amount of Dkk-1 or GSK3*β* within TG and BG had no difference (*P* > 0.05), as well as within GG and NG (*P* > 0.05). But in TG or BG, these amounts were lower than those in GG or NG (*P* < 0.05). So, *β*-catenin, as the intracellular signal transducer of Wnt/*β*-catenin pathway, its expression were significantly higher in BG or TG (no difference between these two groups, *P* > 0.05) than those in GG or NG (no difference between these two groups, *P* > 0.05). The expression of osteogenesis marker, Runx2, was also more upregulated (*P* < 0.05) in BG or TG than that in GG or NG. On the contrary, the adipogenesis marker, PPAR*γ*, had the opposite expression: lower in BG or TG and higher in GG or NG. Also, the expressions of Dkk-1, GSK3*β*, *β*-catenin, Runx2, and PPAR*γ* had no statistical significances (*P* > 0.05) between BG and TG or between GG and NG (NS, *P* > 0.05; ^∗^ *P* < 0.05).

The RNA and protein expression of GSK-3*β* and *β*-catenin, members of the canonical Wnt pathway, and Dkk-1, the extracellular antagonist of this cascade, were assessed by RT-PCR and Western blot, respectively. The expression of GSK-3*β* and *β*-catenin was significantly lower and higher, respectively, in BG and TG than in the other two groups (Figures 2(B) and 2(G); Figures 2(C) and Figure 2(H); *P* < 0.05). The expression of Dkk-1 (Figures 2(A) and 2(F)) was also significantly lower in BG and TG (*P* < 0.05) than in the other two groups. By contrast, Runx2 and PPAR*γ* (Figures 2(D) and 2(I); Figures 2(E) and 2(J)), globally accepted markers of osteogenesis or adipogenesis, respectively, exhibited opposing patterns of expression. Runx2 expression was significantly higher in BG and TG than in GG and NG (*P* < 0.05), whereas PPAR*γ* expression was significantly lower in BG and TG than in GG and NG (*P* < 0.05) (Figure 2).

### 3.3. In Vivo H&E Staining of the Femoral Heads of Rats

**Figure 3:**
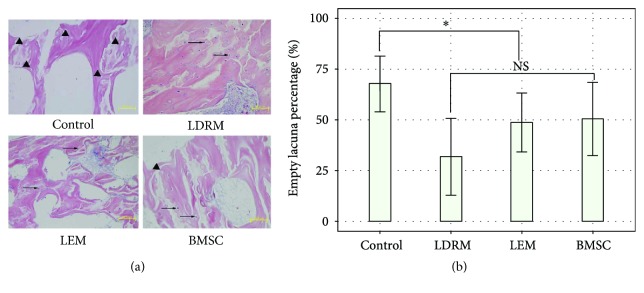
H&E staining of femoral head specimens from 4 groups of rats (a) and the statistical data for the empty lacuna rate (b). Thinner trabecula and higher mean empty lacuna (black triangle) percentage in the control group (67.7% ± 13.8%, *P* < 0.05) than that in others. Normal trabecula, more living osteocytes (black arrow), and lower empty lacuna percentage (31.8% ± 19.0% in LDRM, 48.7% ± 14.6% in LEM, and 50.4% ± 18.1% in BMSC; *P* > 0.05) in the rest of the three groups (scale bar = 50 *μ*m. NS, *P* > 0.05; ^∗^ *P* < 0.05).

Eight weeks after the operation, all 80 rats were sacrificed, and H&E staining was performed. The diagnostic criterion of ONFH was empty lacunae. Briefly, ten fields (20x) from an H&E staining section of one rat were randomly selected to count empty lacunae. If empty lacunae were found in over 5 fields (empty lacunae percentage > 50%), ONFH was diagnosed in a rat. In the control group, most of the trabeculae were cracked, discontinuous, and reduced, and the mean percentage of empty lacunae was 67.7% ± 13.8%, confirming the presence of ONFH. These histological phenomena were observed in 18 of 20 rats, corresponding to an incidence of ONFH of 90% in control group. In the LDRM group, normal, continuous trabeculae were observed in 13 rats, with an empty lacunae percentage far below 50%; ONFH was identified in only 7 rats, corresponding to an overall incidence of 35%. The mean percentage of empty lacunae in all 20 rats was 31.8% ± 19.0%. By comparison, 55% (11/20) of the rats in the LEM group developed ONFH, with a mean empty lacuna percentage of 48.7% ± 14.6%; in the BMSC group, the incidence of ONFH was 50% (10/20), and the mean percentage of empty lacunae was 50.4% ± 18.1%. The differences in ONFH incidence and the mean percentage of empty lacunae between the control group and the other groups were statistically significant (*P* < 0.05). However, the differences among the other three groups were not significant (*P* > 0.05) (Figure 3 and Table 1).

### 3.4. In Vivo Oil Red O Staining of the Femoral Head

**Figure 4:**
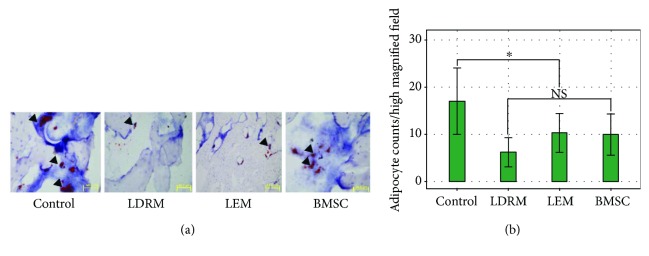
In vivo Oil Red O staining of femoral heads from rats (a) and the measured data (b). The control group contained significantly more adipocytes (black triangle) per high magnified filed (17.05 ± 7.03, *P* < 0.05) than the other groups (6.21 ± 3.06, 10.32 ± 4.12, and 9.97 ± 4.35 in LDRM, LEM, and BMSC, resp., *P* > 0.05), and the cell size was also larger in the control group than that in others (scale bar = 50 *μ*m. NS, *P* > 0.05; ^∗^ *P* < 0.05).

Eight weeks after the operation, Oil Red O staining was performed to visualize adipocytes in femoral head samples from the 4 groups. Red-stained cells in each rat specimen were counted under three different 40x fields. The number of red-stained cells was highest in the control group, with a mean of 17.05 ± 7.03, followed by 6.21 ± 3.06, 10.32 ± 4.12, and 9.97 ± 4.35 in the LDRM, LEM, and BMSC groups, respectively. Only the differences between the control group and the other three groups were significant (*P* < 0.05) (Figure 4).

### 3.5. In Vivo Immunohistochemical Analyses of the Expression of Dkk-1, PPAR*γ*, GSK-3*β*, Runx2, and *β*-Catenin in the Femoral Head

**Figure 5:**
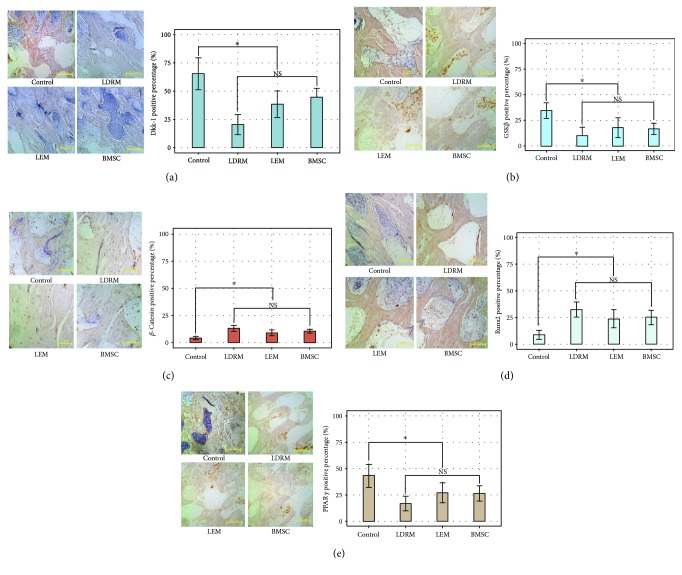
Immunohistochemical analyses for expression of Dkk-1(a), GSK3*β* (b), *β*-catenin (c), Runx2 (d), or PPAR*γ* (e) in femoral head specimens from 4 group rats. Dkk-1 positive percentage in the control group was stronger than the rest, while the positive rate in LDRM was significantly weaker than LEM or BMSC. GSK-3*β* positive. Similarly, GSK3*β* positive rate in the control group was significantly stronger than others (*P* < 0.05). Positive rate of *β*-catenin or Runx2 was significantly higher in LDRM, LEM, or BMSC (but *P* > 0.05 within these three) than the control group (*P* < 0.05). PPAR*γ*, with its strongest positive rate in the control group, was significantly higher than the LDRM, LEM, or BMSC (*P* > 0.05 within these three as well) (scale bar = 50 *μ*m. NS, *P* > 0.05; ^∗^ *P* < 0.05).

The expression of five proteins, Dkk-1, PPAR*γ*, GSK-3*β*, Runx2, and *β*-catenin, in the rat femoral head, was immunohistochemically analyzed, and the number of positive cells was quantified by Image-Pro Plus (ver. 6.0). The percentage of Dkk-1-positive cells was significantly (*P* < 0.05) higher in the control group (mean positive rate of 65.41% ± 14.07%) than in the other groups (LDRM, 20.35% ± 8.80%; LEM, 38.98% ± 11.69%; BMSC, 44.66% ± 7.83%). In addition, the Dkk-1-positive staining intensity was significantly lower in LDRM than in either LDRM or BMSC (*P* < 0.05). Similarly, the percentage of PPAR*γ*- or GSK-3*β*-positive cells was significantly (*P* < 0.05) higher in the control group (43.14% ± 10.85% and 34.54% ± 7.77%, resp.) than in the other three groups (LDRM, 16.77% ± 6.93% and 10.08% ± 8.14%; LEM, 27.08% ± 9.43% and 17.70% ± 9.66%; BMSC, 26.45% ± 7.22% and 16.68% ± 5.45%). By contrast, the percentages of positive staining for Runx2 and *β*-catenin were significantly (*P* < 0.05) lower in the control group (8.78% ± 4.30% and 4.21% ± 1.45%, resp.) than in the other three groups. Positive staining for Runx2 and *β*-catenin was highest in LDRM (32.50% ± 7.12% and 13.13% ± 2.77%), but these values did not differ significantly (*P* > 0.05) from those in LEM (23.74% ± 8.45% and 9.05% ± 2.64%) or BMSC (25.01% ± 6.67% and 10.51% ± 1.70%) (Figure 5).

## 4. Discussion

Although clinical success in BMSC implantation has been reported, the treated ONFH cases were heterogeneous in etiology [3, 6, 21]: trauma, glucocorticoid use, alcohol abuse, and idiopathic. This etiological heterogeneity may reduce the validity of these results due to low osteogenesis or proliferation of autologous BMSCs in patients with GC-induced ONFH [10–12]. Hence, we hypothesized that implanting BMSCs from healthy individuals in those with GC-induced ONFH might produce superior outcomes compared to implantation of autologous cells.

Moreover, there are new therapeutic strategies for ONFH involving modifications of genes encoding stem cell-generating growth factors, such as bone morphogenetic protein-2 (BMP-2) and vascular endothelial growth factor (VEGF) [22–24]. Tang and colleagues [18] repaired surgically induced ONFH in goats by introducing exogenous BMP-2 in BMSCs. In another study, Hang [19] and colleagues successfully induced bone regeneration in ONFH animals by implanting BMSCs transfected with VEGF. Our previous studies [13–16] suggested that GC-induced extracellular Dkk-1 upregulation and the resultant interruption of intracellular Wnt/*β*-catenin transduction not only reduce the differentiation and lifespan of osteoblasts or osteocytes but also alter the osteogenesis of BMSCs toward adipogenesis. Our findings and those of others [17, 25, 26] rationally support a potential role of these abnormalities in the pathogenesis of GC-induced ONFH. Consistent with this role, inhibition of Dkk-1 shifts adipogenesis [14] toward osteogenesis and promotes bone formation [27]. Thus, it is reasonable to propose the implantation of Dkk-1 RNAi-modified BMSCs to simultaneously prevent the onset of GC-induced ONFH and reverse its progress.

In vitro, glucocorticoid (GC) treatment induced the upregulation of Dkk-1 at both the mRNA and protein levels. This increase in Dkk-1 expression resulted in activation of GSK-3*β*, a downstream inhibitory factor for *β*-catenin, and in turn, downregulation of *β*-catenin. GSK-3*β* and *β*-catenin are members of the canonical Wnt signaling pathway, consistent with previous suggestions of suppression of this pathway via enhancement of Dkk-1 by GC treatment as a possible mechanism of onset of GC-induced ONFH. Reduction of Dkk-1 expression by RNAi blocked its subsequent anti-Wnt/*β*-catenin effects induced by GC and shifted the differentiation of BMSCs from adipocytes toward osteoblasts.

In vivo, H&E, Oil Red O, and immunohistochemical staining revealed less GC-induced ONFH development in the treated groups (LDRM, LEM, and BMSC), as evidenced by fewer empty lacunae, a lower percentage of bone necrosis, and less adipogenesis compared with the untreated group (control). However, the differences between the three treated groups, LDRM and LEM, were not statistically significant, in contrast to the significant differences in mRNA and protein expression and Oil Red O staining between TG and NG in vitro. Also, the in vivo results revealed that LDRM group was not significantly improved, compared with the BMSC group whose GC-induced rats treated with normal and unmodified BMSCs. Thus, although BMSCs modified by lentivirus-meditated Dkk-1 RNAi were able to activate the Wnt/*β*-catenin pathway, inhibit continuous GC-induced adipogenesis, and maintain osteogenesis in vitro, their ability to arrest the progress of GC-induced ONFH in vivo was not significantly superior to that of negative control-transfected or even unmodified BMSCs (BMSC group).

The discrepancies in the results of the in vitro and in vivo studies may be attributed to differences in protocol. In vitro, we first reduced Dkk-1 expression in BMSCs by introducing lentivirus-meditated RNAi and then subsequently exposed the cells to GC to assess the protective effects of this modification. Performing transfection prior to GC exposure is a common sequence in studies of the effects of gene modifications in the pathogenesis of GC-induced ONFH [28]. For example, Yun and coworkers [29] firstly silence the expression of GSK-3*β* in osteoblasts by siRNA transfection, and then the cells were treated with Dex to assess that whether osteoblasts with GSK-3*β* silence could resist to the Dex-induced apoptosis. Likewise, Butler et al. [30] also knocked down the expression of Dkk-1 in osteoblasts in the first place and then exposed the cells to Dex.

However, the protocols of studies of stem cell therapies for GC-induced ONFH differ from those of pathogenesis research. In general, GC-induced ONFH animal models are established [31, 32] or patients are recruited, both prior to the stem cell treatments, with no subsequent GC exposure in the remainder of the study. Wen and workmates [32] established the GC-induced ONFH rabbits with a combination of LPS (10 *μ*g/kg body weight, one injection) and MPS (20 mg/kg body weight, three times injection) injection in the first place and then transplanted BMSCs which were hepatocyte growth factor (HGF)-overexpressed to treat this disease. Ding et al. [33] also used the similar treated process: inducing ONFH animal model firstly and then the Hif-1*α* transgenic BMSCs were transplanted to the necrotic site.

In vitro, GC-induced Dkk-1 activation clearly impaired the Wnt/*β*-catenin pathway, with an increase in adipogenesis and decrease in osteogenesis; only the group in which Dkk-1 was downregulated, BMSC, escaped this fate. However, although studies have demonstrated that deletion of Dkk-1 significantly increases Wnt/*β*-catenin expression and bone formation in vivo [27], a corresponding increase in Wnt/*β*-catenin expression was not observed in the group treated with BMSCs in which Dkk-1 was downregulated (LDRM) compared with the groups that received BMSCs without genetic modification (LEM and BMSC). This discrepancy with the previous report [27] indicates further study. A gene therapy pattern reported by Zhang et al. [31] could be helpful. After establishing GC-induced ONFH rabbit model, Zhang and colleagues directly injected adeno-associated virus vector which carried both VEGF and BMP genes into the femoral head via core decompression tunnel.

The present study is subject to limitations that may reduce the validity of our conclusions. First, the bone mass of the femoral head, an indicator of new bone formation, was not quantified in each treated group. Second, the implanted cells were allogeneic BMSCs. Although transplantation of allogeneic BMSCs has been used previously to treat patients with ONFH [34] or other joint diseases [35–37], the immunological safety of these allogeneic BMSCs remains unclear [38–40]. Further studies are necessary.

## 5. Conclusion

BMSCs with lentivirus-meditated Dkk-1 RNAi not only prevented the GC-induced decrease in the Wnt/*β*-catenin cascade, normalized the expression of the osteogenic marker Runx2, and attenuated the adipogenic marker PPAR*γ* but also maintained osteogenesis rather than adipogenesis under GC exposure. Moreover, the implantation of these Dkk-1-downregulated BMSCs into the necrotic femoral head in rats with GC-induced ONFH prevented progression of the disease, although the effects were not significantly superior to treatment with nongenetically modified BMSCs.

## Figures and Tables

**Table 1:** Incidence of GC-induced ONFH in each group.

Group	GC-induced ONFH		
	Yes	No	Total
Control^∗∗^	18	2	20
LDRM^∗^	7	13	20
LEM^∗^	11	9	20
BMSC^∗^	10	10	20

^∗^ *P* > 0.05 for comparisons among LDRM, LEM, and BMSC. ^∗∗^ *P* < 0.05 compared to the other three groups.

## Supplementary Materials

Supplementary Materials Figure 1: BMSC morphological observation (a), phenotypic analyses (b), and osteogenetic and adipogenic induction (c and d). Figure 2: full workflow of constructing the lentivirus-meditated vector and negative controlled counterpart. Figure 3: Transfection effect and optimal MOI analyses. From A to E, MOI: 0, 15, 25, 35, and 45. Figure 4: the protocol of GC-induced ONFH establishment and the cell implantations. Figure 5: H&E staining of femoral heads in GC-induced ONFH rats (a: GC group; b: normal controlled group). GC group with thinner trabecula and significantly more empty lacuna. Scale bar = 50.0 *μ*m.
