# Supplementary material for: Efficiency of Cell Therapy to GC-Induced ONFH: BMSCs with Dkk-1 Interference Is Not Superior to Unmodified BMSCs
Source: Stem Cells Int. 2018 May 22;2018:1340252. doi: 10.1155/2018/1340252 (PMC5987233; doi:10.1155/2018/1340252)
Supplement: Supplementary Materials — Figure 1: BMSC morphological observation (a), phenotypic analyses (b), and osteogenetic and adipogenic induction (c and d). Figure 2: full workflow of constructing the lentivirus-meditated vector and negative controlled counterpart. Figure 3: Transfection effect and optimal MOI analyses. From A to E, MOI: 0, 15, 25, 35, and 45. Figure 4: the protocol of GC-induced ONFH establishment and the cell implantations. Figure 5: H&E staining of femoral heads in GC-induced ONFH rats (a: GC group; b: normal controlled group). GC group with thinner trabecula and significantly more empty lacuna. Scale bar = 50.0 μm. [file 1340252.f1.docx]

Supplementary data
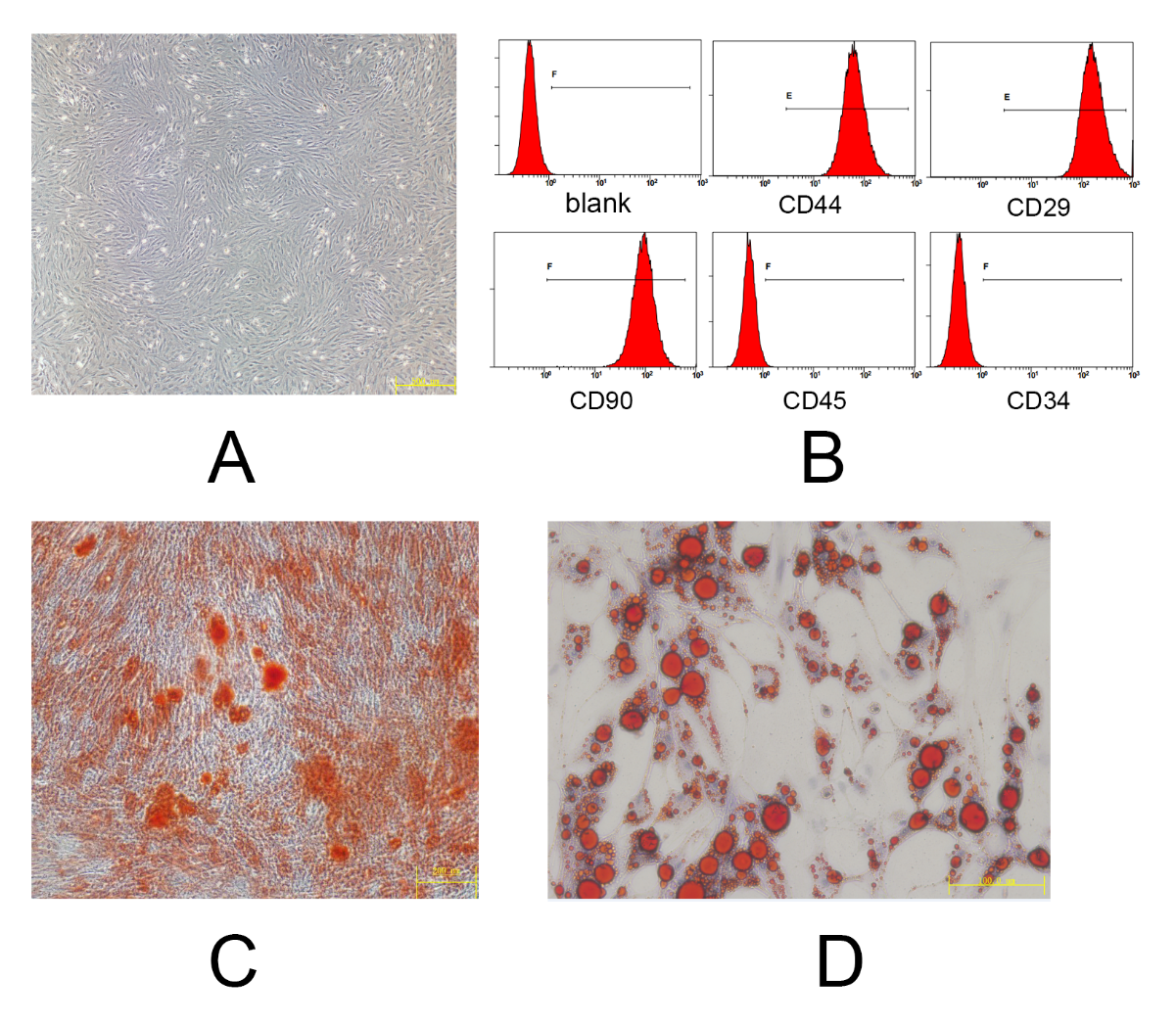


Figure 1 BMSCs morphological observation(A), phenotypic analyses(B), osteogenetic and adipogenetic induction (C and D)


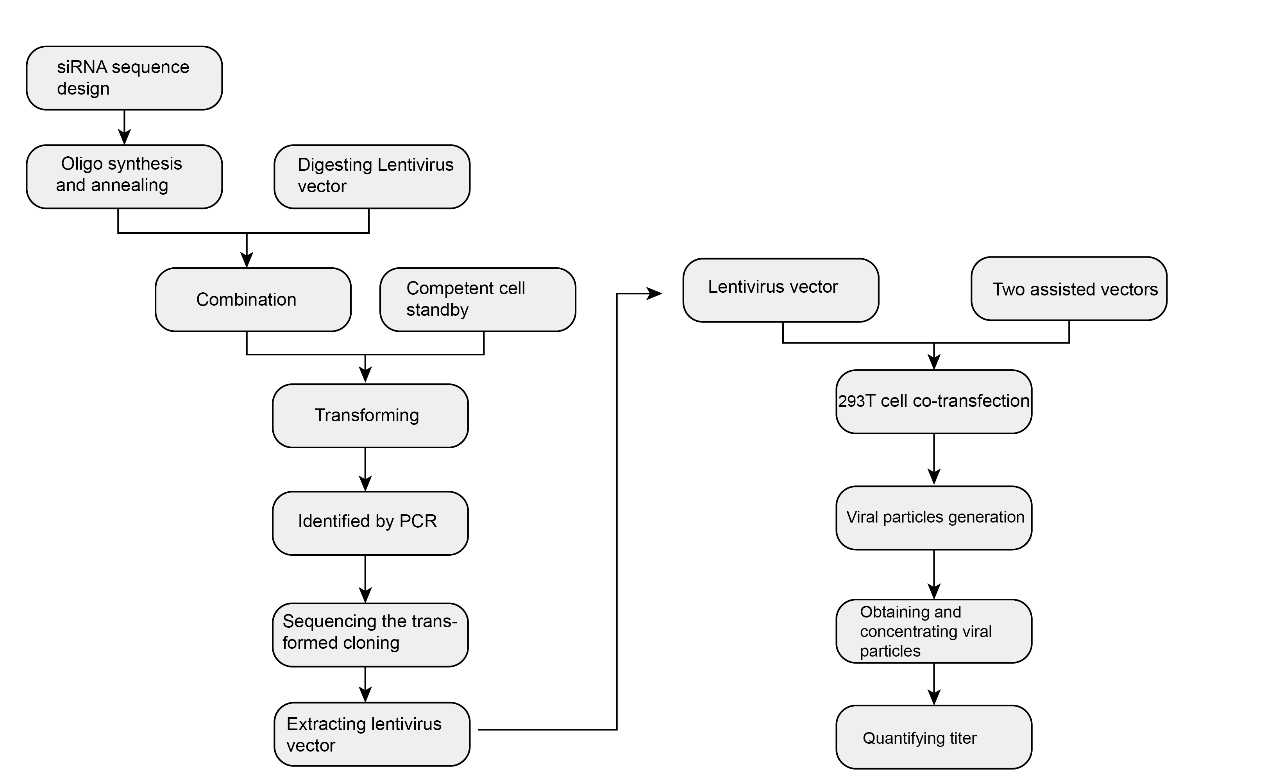


Figure 2 Full work-flow of constructing of lentivirus-meditated vector and negative controlled counterpart


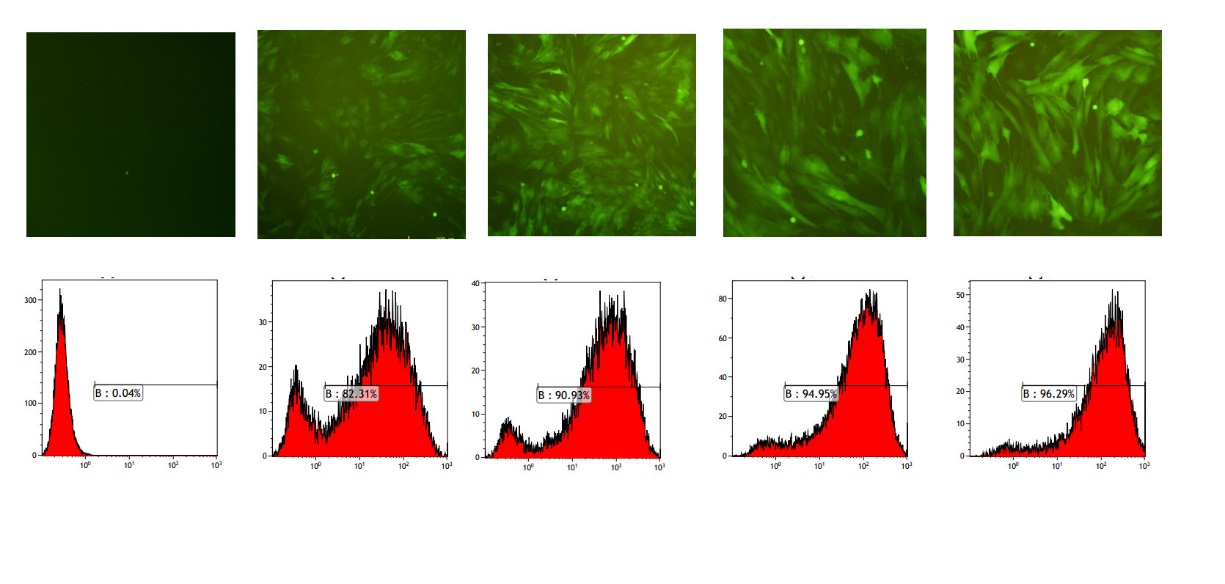


Figure 3 Transfection effect and optimal MOI analyses. From left to right, MOI from 0, 15,25,35 and 45.

Table 1 Primers informations

| Gene | Primer sequences |
| --- | --- |
| *Ctnnb1* | F：5’ GGAAAGCAAGCTCATCATTCTG 3’ |
|  | R：5’ AGTGCCTGCATCCCACCAGCTT 3’ |
| *Runx2* | F: 5’ GGACGAGGCAAGAGTTTCACTT3’ |
|  | R：5’ CT GTCTGTGCCT TC TTGGTTCC3’ |
| *Dkk-1* | F: 5’ TATCACACCAAAGGGCAAGAAG3’ |
|  | R：5’ TGATGGTGAT CT T TCTGT ATCC 3’ |
| *Gsk-3β* | F: 5’ CACAGAACCTCTTGCTGGATCC 3’ |
|  | R：5’ GGTGCCCTGTAGTACCGAGAAC3’ |
| *Pparg* | F：5’ GTCTCACAATGCCATCAGGTTT 3’  R：5’ TTCAGCTGGTCGATATCACTGG 3’ |
| *Gapdh* | F：5' GACATCAAGAAGGTGGTGAAGC 3' |
|  | R：5’ CAGCATCGAAGGTAGAGGAGTG3’ |


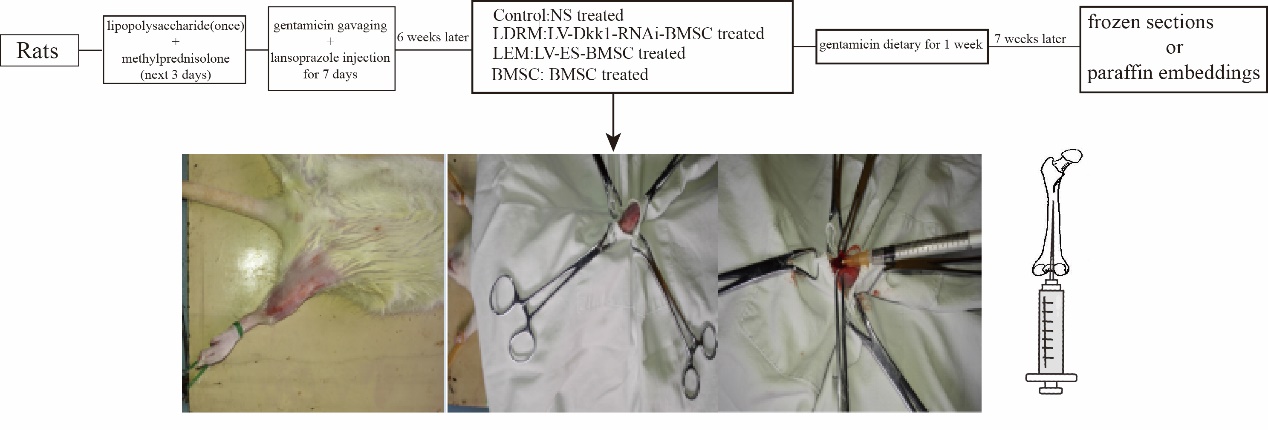


Figure 4 The protocol of GC-induced ONFH establishment and the cell implantations.


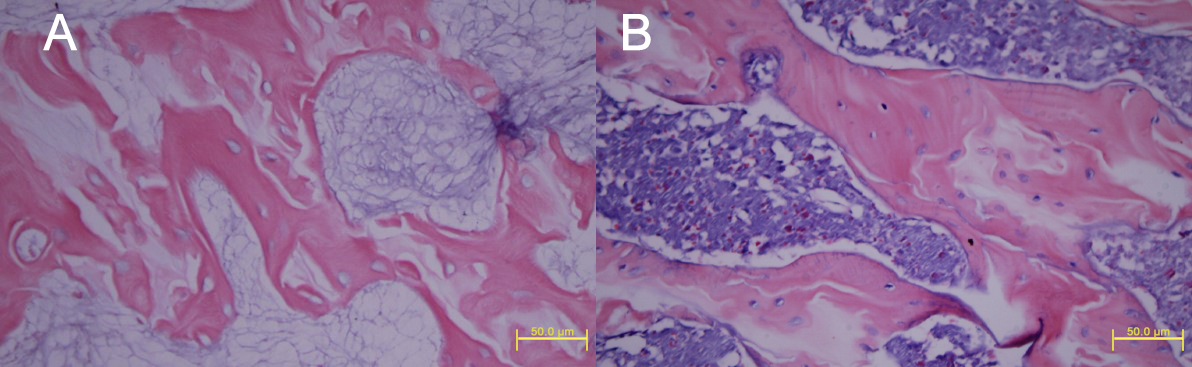


Figure 5 H&E staining of femoral heads in GC-induced ONFH rats (A: GC group; B: normal controlled group). GC group with thinner trabecula and significantly more empty lacuna. Scale bar = 50.0 μm
